# The herbal compound Songyou Yin (SYY) inhibits hepatocellular carcinoma growth and improves survival in models of chronic fibrosis via paracrine inhibition of activated hepatic stellate cells

**DOI:** 10.18632/oncotarget.5313

**Published:** 2015-10-22

**Authors:** Yang Bu, Qing-An Jia, Zheng-Gang Ren, Tong-Chun Xue, Quan-Bao Zhang, Ke-Zhi Zhang, Qiang-Bo Zhang, Yang You, Hui Tian, Lun-Xiu Qin, Zhao-You Tang

**Affiliations:** ^1^ Hepatobiliary Surgery, General Hospital of Ningxia Medical University, Yinchuan 750001, China; ^2^ Liver Cancer Institute, Zhongshan Hospital, Fudan University, Key Laboratory of Carcinogenesis and Cancer Invasion, Ministry of Education, Shanghai 200032, China; ^3^ Cancer Center, Institutes of Biomedical Sciences, General Surgery, Huashan Hospital, Fudan University, Shanghai 200032, China

**Keywords:** Songyou Yin, hepatocellular carcinoma, chronic fibrosis, activated hepatic stellate cells

## Abstract

Chronic fibrosis is a major risk factor for the development of hepatocellular carcinoma (HCC). The pathological progression of hepatic fibrosis has been linked to cellular processes that promote tumor growth and metastasis. Several recent studies have highlighted the cross-talk between tumor cells and activated hepatic stellate cells (aHSCs) in HCC. The herbal compound Songyou Yin (SYY) is known to attenuate hepatoma cell invasion and metastasis via down-regulation of cytokine secretion by aHSCs. However the underlying mechanism of SYY treatment in reversal of hepatic fibrosis and metastasis of liver cancers is not known. In the current study, a nude mouse model with liver fibrosis bearing orthotopic xenograft was established and we found that SYY could reduce associated fibrosis, inhibit tumor growth and improve survival. In the subcutaneous tumor model with fibrosis, we found that SYY could inhibit liver cancer. *In vitro*, hepatoma cells incubated with conditioned media (CM) from SYY treated aHSCs showed reduced proliferation, decrease in colony formation and invasive potential. SYY treated group showed altered gene expression, with 1205 genes up-regulated and 1323 genes down-regulated. Gene cluster analysis indicated that phosphatidylinositol-3-kinase (PI3K) was one of the key genes altered in the expression profiles. PI3K related markers were all significantly down-regulated. ELISA also indicated decreased secretion of cytokines which were regulated by PI3K/AKT signaling after SYY treatment in the hepatic stellate cell line, LX2. These data clearly demonstrate that SYY therapy inhibits HCC invasive and metastatic potential and improves survival in nude mice models with chronic fibrosis background via inhibition of cytokine secretion by activated hepatic stellate cells.

## INTRODUCTION

Liver cancer (mainly hepatocellular carcinoma [HCC]), ranks as the fifth most common cancer and worldwide it is the second most frequent cause of death in men [1]. In China, it is observed that chronic hepatitis followed by concomitant liver cirrhosis is the predominant risk factor for the development of HCC [2]. A previous study has demonstrated that activated hepatic stellate cells (aHSCs) play a key role in causing liver cirrhosis and infiltration into the HCC stroma, which supports malignancy progression [3].

Some reports have shown that in response to liver damage from viruses, alcohol, drug abuse and any other cause, the aHSCs trans-differentiate into myofibroblasts (MFs), which leads to hepatic fibrosis or even cirrhosis [4, 5]. Several recent studies have indicated that cross-talk occurs between tumor cells and aHSCs during progression of HCC [6, 7]. Wang and colleagues [8] reported that cancer associated fibroblasts (CAFs) isolated from lung cancer tissue produces hepatocyte growth factor (HGF), which activates the c-Met pathway resulting in the invasion and metastasis of cancer cells [9]. Another study reported that fibroblasts isolated from breast cancer tissue enhances cancer cell invasiveness through an interleukin-6 (IL-6)-dependent signaling mechanism [10]. Thus, there is evidence to support the concept that aHSCs contribute to the development and progression of different types of malignancies. Similarly, aHSCs enhance hepatoma cell invasiveness and metastasis through paracrine signaling mechanisms involving cytokine secretion [11, 12]. Furthermore, the transforming growth factor-β (TGF-β) receptor inhibitor, LY2109761, was shown to inhibit the production of TGF-β secretion by CAFs, which blocked the cross-talk between CAFs and HCC and inhibited progression of HCC [13]. Supported by these previous studies, aHSCs are recognized critical players in the development and progression of malignancy [14, 15]. Inhibition of aHSC activity in the tumor microenvironment represents a potential therapeutic strategy to prevent and treat HCC.

Since ancient times, herbal medicines have been used in China to treat different malignancies. [16]. Several studies have shown that extracts from traditional herbal medicines possess anticancer potential, warranting further study. These studies have shown that herbal extracts possess anticancer activity and can modulate cell cycle, apoptotic signaling, expression of angiogenic factors, invasion and metastatic potential in cancer cells [17–27]. The herbal medicine Bu-Zhong-Yi-Qi Tang is a mixture of ten herbs, and was shown to suppress the growth of hepatoma cells *in vitro* through the activation of a p53-independent pro-apoptotic signaling mechanism [28]. Similarly, Sho-Saiko-To, which is a mixture of seven herbs, inhibited the proliferation of human hepatoma cells by inducing cell cycle arrest and apoptosis [29]. Our recent study with Songyou Yin (SYY), a mixture of five herbs, showed inhibition of HCC growth and prolonged survival in a mice model by inducing caspase-3-dependent tumor cell apoptosis [30]. We also demonstrated that SYY inhibited HCC invasiveness by down-regulation of enzyme matrix metalloproteinase-2 (MMP-2) [30]. Xiong *et al*., found that SYY could attenuate oxaliplatin-enhanced HCC metastatic potential [31]. Jia *et al*., reported that SYY attenuated hepatoma cell invasiveness and metastasis *in vitro* via down-regulation of cytokines secreted by aHSCs [11]. However, whether SYY has a role in reversal of hepatic fibrosis and which signal transduction pathway leads to inhibition of hepatoma progression remains largely unknown.

In the present study, a mouse model with a fibrotic background was established to determine if SYY could attenuate hepatic fibrosis and block the cross-talk between aHSCs and HCC in xenograft tumors. We also investigated the ability of SYY to indirectly influence the malignancy potential and progression of hepatoma cells and the molecular mechanisms involved.

## RESULTS

### Establishment of a nude mouse model with fibrosis

**Figure 1:**
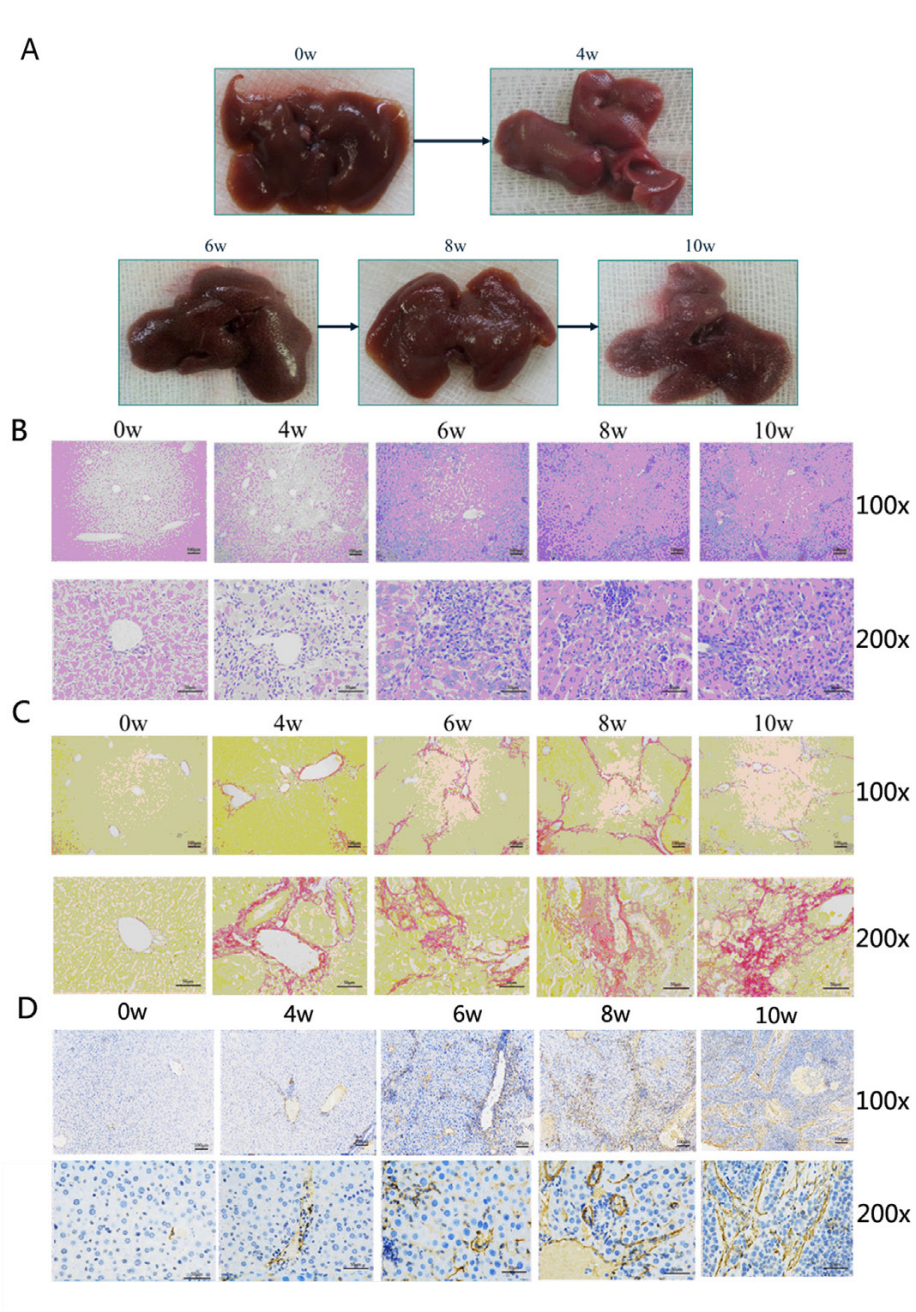
The nude mouse model with cirrhosis induced by carbon tetrachloride (CCL_4_) was successfully established **A.** The increase in severity of hepatic fibrosis was observed as the presence of more number of particles on the liver surface with reduction in liver volume with the prolonged treatment with CCL_4_. **B.** H&E staining exhibited the same tendency of increased f hepatic fibrosis with increased fibrous connective tissue. **C.** Sirius staining also showed continuous collagen accumulation induced by prolonged CCL_4_ treatment. **D.** α-SMA, which is a marker of hepatic fibrosis, was also up-regulated after treatment with CCl_4_.

In order to study the correlation between liver fibrosis and HCC, it is important to establish a stable mouse model with fibrosis. We utilized the method of subcutaneous injection of CCl_4_ and found, as expected, that the severity of hepatic fibrosis increased with the prolonged treatment of CCl_4_. The liver stiffness, the particles on the liver surface and volume are three of the important exterior characteristics in evaluating liver cirrhosis. We found the severity of hepatic fibrosis was increased by exhibiting the increased particles on the liver surface, and there was reduced liver volume with the prolonged treatment of CCl_4_ (Figure 1A). H&E and Sirius staining showed there was continuous collagen accumulation induced by prolonged CCl_4_ treatment (Figure 1B, 1C). α-SMA, which is a marker of hepatic fibrosis, was also up-regulated after CCl_4_ treatment (Figure 1D).

### SYY inhibited tumor growth and reduced associated fibrosis in nude mice bearing orthotopic xenografts with a fibrosis background

**Figure 2:**
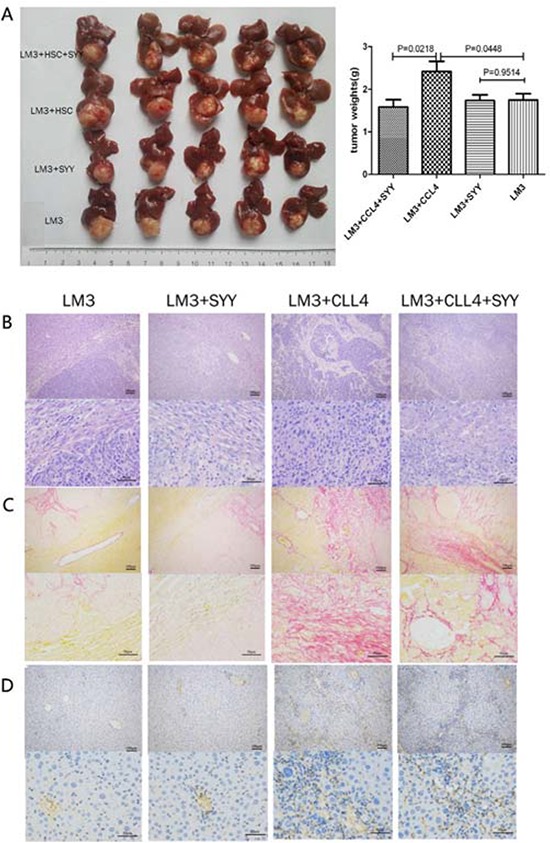
The nude mouse model bearing orthotopic xenografts was established The orthotopic tumors in the models with cirrhosis background were larger than tumors in the models with normal liver tissue background. Treatment of tumors with cirrhosis background with SYY significantly reduced tumor volume. **A.** There were no significant changes in tumor volume with normal liver tissue background. **B.** H&E staining exhibited reduced fibrous connective tissue in the tumor stroma. **C.** Sirius staining also showed that reduced collagen accumulation and **D.** down-regulated expression of α-SMA in tumor stroma were all related to SYY treatment.

Based on the nude mouse model with fibrosis mentioned above, we further established the nude mouse model bearing orthotopic xenograft with fibrosis. These were divided in untreated and SYY treated groups. There was enhanced proliferation in the untreated group (HCCLM3 + CCl_4_ 2.418 ± 0.24 *vs*. HCCLM3 1.748 ± 0.15, *P* = 0.0448). SYY (2 g/kg/day) exhibited no significant inhibition of tumor growth (HCCLM3, 1.74 8 ± 0.15 *vs*. HCCLM3 + SYY 1.736 ± 0.13, *P* = 0.9514), while, in the treated group induced by CCl_4_, the same dosage of SYY showed a significant inhibition of tumor growth (HCCLM3 + CCl_4_ 2.418 ± 0.2376 *vs*. HCCLM3 + CCl_4_ + SYY 1.584 ± 0.1725, *P* = 0.0218) (Figure 2A). H&E and Sirius staining highlighted the increased fibrous connective tissue in tumor stroma induced by CCl_4_ (Figure 2B, 2C). The expression of α-SMA, also increased (HCCLM3 1259 ± 112.2 *vs*. HCCLM3 + CCl_4_ 12180 ± 1073, *P* = 0.0006) in untreated group, but was down-regulated in SYY treated group (HCCLM3 + CCl_4_ 12180 ± 1073 *vs*. HCCLM3 + CCl_4_ + SYY 7327 ± 476.3, *P* = 0.0144) (Figure 2D).

### SYY inhibited HCC growth, reduced associated fibrosis and prolonged survival in the xenograft tumor model with fibrosis background

**Figure 3:**
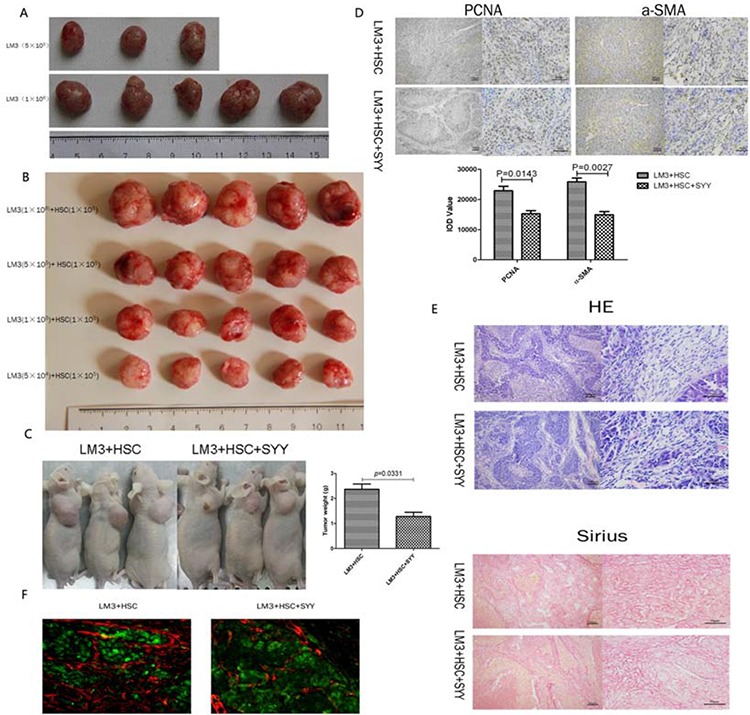
Subcutaneous tumor model of liver cancer was established and the correlation among tumor parenchymal cells, aHSCs in the stroma and SYY were investigated LM3 in the range of 5 × 10^4^ to 1 × 10^5^ cells did not form tumors, and even with the total number of cells approaching 5 × 10^5^ only half of the amount of tumors were formed **A.** LM3 in the number of 5 × 10^4^ can form tumors when added with 1 × 10^5^ aHSCs. The volumes of tumors were related to the number of LM3 cells **B.** Thus, 5 × 10^5^ LM3 cells with 5 × 10^5^ aHSCs were chosen to form tumors and the tumor volume was significantly reduced after treatment with SYY **C.** PCNA and α-SMA were both down-regulated in tumor tissues after treatment with SYY **D.** HE and Sirius staining exhibited reduced fibrous connective tissue and collagen accumulation in tumor tissues after treatment with SYY **E.** Finally, aHSCs, which were marked by α-SMA, were significantly decreased, as evident by immunofluroescence **F.**

**Figure 4:**
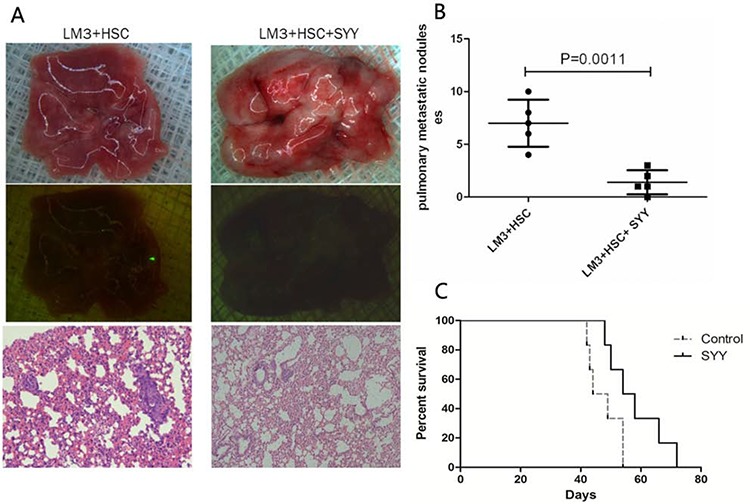
Reduced lung metastasis and prolonged survival were found in the orthotopic nude mouse models with cirrhosis background after treatment with SYY

The nude mouse xenograft model with a fibrosis background was established and the correlation between tumor parenchymal cells and aHSCs was evaluated. The HCCLM3 cell density of 5 × 10^4^ and 1 × 10^5^ could not form xenograft tumors. Even with the total number of cells approaching 5 × 10^5^, only half of the xenograft tumors were formed. While HCCLM3 with cell density of 1 × 10^6^ could form full xenograft tumors (Figure 3A, Table 1). The mixture of 5 × 10^4^ HCCLM3 and 1 × 10^5^ aHSCs, formed xenograft tumors. The volume of the xenograft tumors were related to the number of HCCLM3 cells used (Figure 3B, Table 1). For further studies, mixture of 5 × 10^5^ HCCLM3 cells and 5 × 10^5^ aHSCs cells was used to form xenograft tumors. There was significant reduction in the tumor volume after treatment with SYY (HCCLM3 + HSC 2.36 ± 0.21 *vs*. HCCLM3 + HSC + SYY 1.12 ± 0.17, *P* = 0.0331) (Figure 3C). PCNA, which is a marker of proliferation, was down-regulated in tumor tissues in SYY treated group (HCCLM3 + HSC 22880 ± 1510 *vs*. HCCLM3 + HSC + SYY 15240 ± 1050, *P* = 0.0143). The expression of α-SMA was also down-regulated after treatment with SYY in the LM3 and HSC mixed tissues (HCCLM3 + HSC 25830 ± 1269 *vs*. HCCLM3 + HSC + SYY 14910 ± 1057, *P* = 0.0027) (Figure 3D). H&E and Sirius staining indicated reduced fibrous tissue and collagen accumulation in tumor tissues after SYY treatment (Figure 3E). Finally, in aHSCs, α-SMA was significantly decreased as demonstrated by immunofluorescence (red staining) (Figure 3F). Meanwhile, the SYY treatment group in the xenograft model showed that hepatic cancer cells were attenuated in metastasizing to the lungs (HCCLM3 + HSC 7.0 ± 1.0 *vs*. HCCLM3 + HSC + SYY 1.4 ± 0.51, *P* = 0.0011) and led to prolonged survival (HCCLM3 + HSC 51.33 ± 5.26 days *vs*. HCCLM3 + HSC + SYY 63.17 ± 9.22 days, *P* = 0.0016) (Figure 4).

**Table 1:** aHSCs promote the growth of HCC

HCCLM3	LX2 (1 × 10^5^ / 0.1ml)	
	(−)	(+)
1 × 10^6^ / 0.1ml	5/5	5/5
5 × 10^5^ / 0.1ml	3/5	5/5
1 × 10^5^ / 0.1ml	0/5	5/5
5 × 10^4^ / 0.1ml	0/5	5/5

### Hepatoma cells treated with CM from SYY pretreated aHSCs showed reduced malignant potential

**Figure 5:**
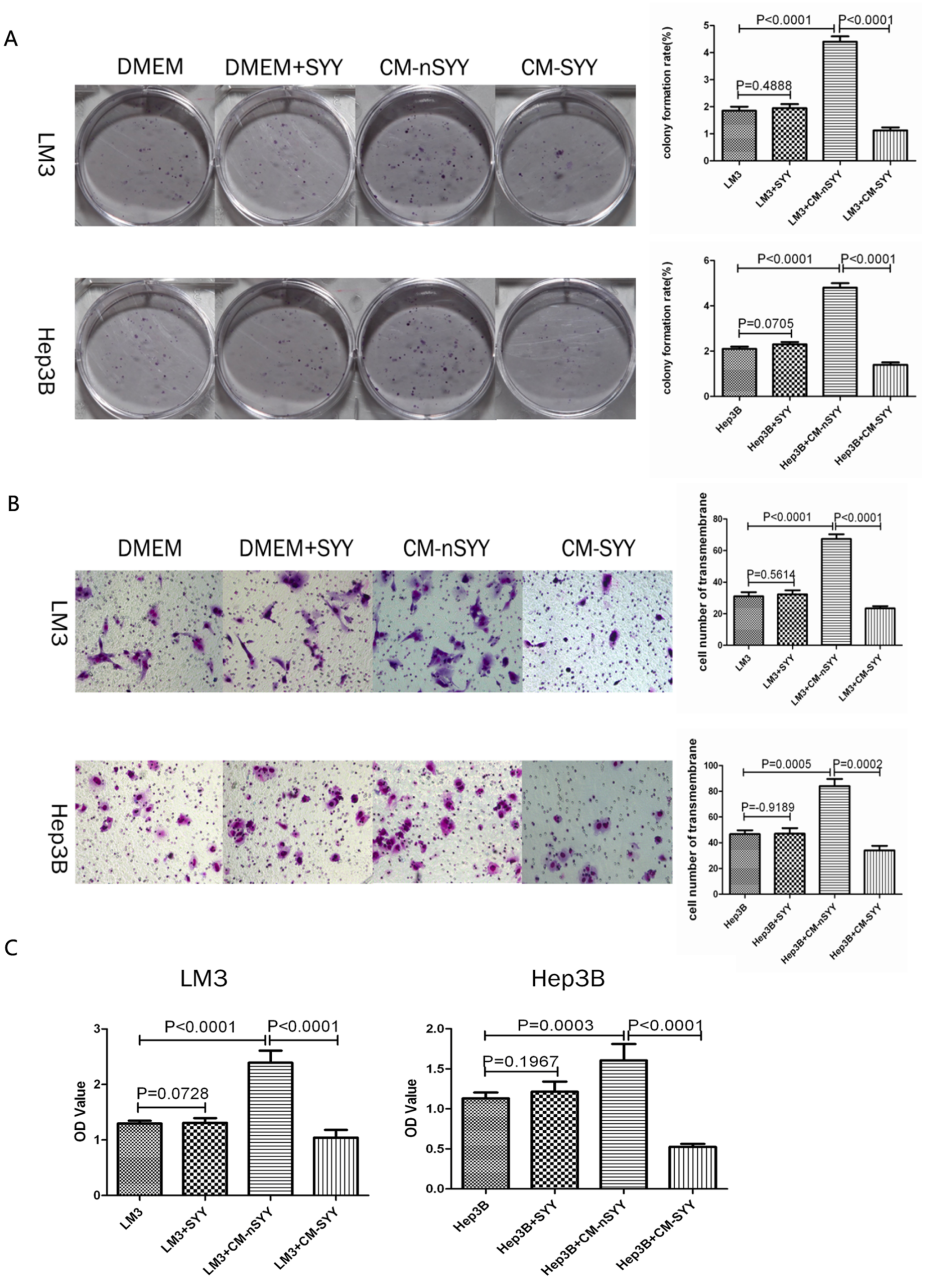
Clonogenic capacity of LM3 and Hep3B cells was enhanced after treatment with CM from aHSCs **A.** The transwell assays demonstrated that LM3 and Hep3B cells treated with CM from aHSCs passed through the basement membrane in larger number than cells in the control group. This effect was reduced by SYY treatment of cells exposed to aHSCs CM **B.** CCK8 assays showed that the proliferative capacity of LM3 and Hep3B was enhanced after treatment with CM from aHSCs, while SYY itself did not promote the proliferation of tumor cells **C.**

While studying the basic characteristics of SYY, we found that SYY exhibited concentration-dependent inhibition. Hence we chose SYY (8 mg/ml) as the final concentration for further experiments, which exhibited no significant cytotoxicity in LX2 (Supplementary materials 1). The clonogenic capacity of HCCLM3 and Hep3B was enhanced after treatment with aHSCs CM (HCCLM3 1.853 ± 0.0867 *vs*. HCCLM3 + CM-nSYY 4.000 ± 0.1155, *P* < 0.0001; Hep3B 2.100 ± 0.0547 *vs*. Hep3B + CM-nSYY 4.800 ± 0.1201, *P* < 0.0001). SYY showed no direct inhibition on the clonogenic capacity of HCCLM3 and Hep3B (HCCLM3 1.853 ± 0.0867 *vs*. HCCLM3 + SYY 1.947 ± 0.0866, *P* = 0.4888; Hep3B 2.100 ± 0.0547 *vs*. Hep3B + SYY 2.300 ± 0.0512, *P* =0.0705). However, the CM from SYY treated aHSCs significantly inhibited the clonogenic capacity of LM3 and Hep3B (HCCLM3 + CM-SYY 4.400 ± 0.1155 *vs*. HCCLM3 + CM-nSYY 1.127 ± 0.0636, *P* < 0.0001; Hep3B + CM-SYY 4.800 ± 0.1201 *vs*. Hep3B + CM-nSYY 1.400 ± 0.0577, *P* < 0.0001) (Figure 5A). The number of HCCLM3 and Hep3B cells passing through the basement membrane was increased after treatment with CM obtained from aHSCs (HCCLM3 31.00 ± 1.528 *vs*. HCCLM3 + CM-nSYY 67.3 3± 1.764, *P* < 0.0001; Hep3B 46.67 ± 1.764 *vs*. Hep3B + CM-nSYY 84.00 ± 3.215, *P* = 0.0005). SYY showed no direct inhibition on the number of HCCLM3 and Hep3B cells passing through the basement membrane (HCCLM3 31.00 ± 1.528 *vs*. HCCLM3 + SYY 32.33 ± 1.453, *P* = 0.5614; Hep3B 46.67 ± 1.764 *vs*. Hep3B + SYY 47.00 ± 2.517, *P* = 0.9189). We did find that the CM from SYY treated aHSCs significantly inhibited the number of HCCLM3 and Hep3B migrating cells (HCCLM3 + CM-nSYY 67.33 ± 1.764 *vs*. HCCLM3 + CM-SYY 23.33 ± 0.8819, *P* < 0.0001; Hep3B + CM-nSYY 84.00 ± 3.215 *vs*. Hep3B + CM-SYY 34.00 ± 2.082, *P* = 0.0002) (Figure 5B).

The cell proliferation of HCCLM3 and Hep3B was enhanced after treatment with CM from aHSCs (HCCLM3 1.296 ± 0.0211 *vs*. HCCLM3 + CM-nSYY 2.391 ± 0.0888, *P* < 0.0001; Hep3B 1.131 ± 0.0301 *vs*. Hep3B + CM-nSYY 1.606 ± 0.0837, *P* = 0.0003). SYY showed no direct inhibition on the proliferative capacity of HCCLM3 and Hep3B (HCCLM3 1.296 ± 0.0211 *vs*. HCCLM3 + SYY 1.310 ± 0.0339, *P* = 0.7280; Hep3B 1.131 ± 0.0301 *vs*. Hep3B + SYY 1.214 ± 0.0521, *P* = 0.1967). While CM from SYY treated aHSCs significantly inhibited the proliferative capacity of HCCLM3 and Hep3B (HCCLM3 + CM-nSYY 2.391 ± 0.0888 *vs*. HCCLM3 +CM-SYY 1.042 ± 0.0569, *P* < 0.0001; Hep3B + CM-nSYY 1.606 ± 0.0837 *vs*. Hep3B + CM-SYY 0.5258 ± 0.0149, *P* < 0.0001) (Figure 5C).

### SYY treatment altered gene expression and cytokine secretion of aHSCs

**Figure 6:**
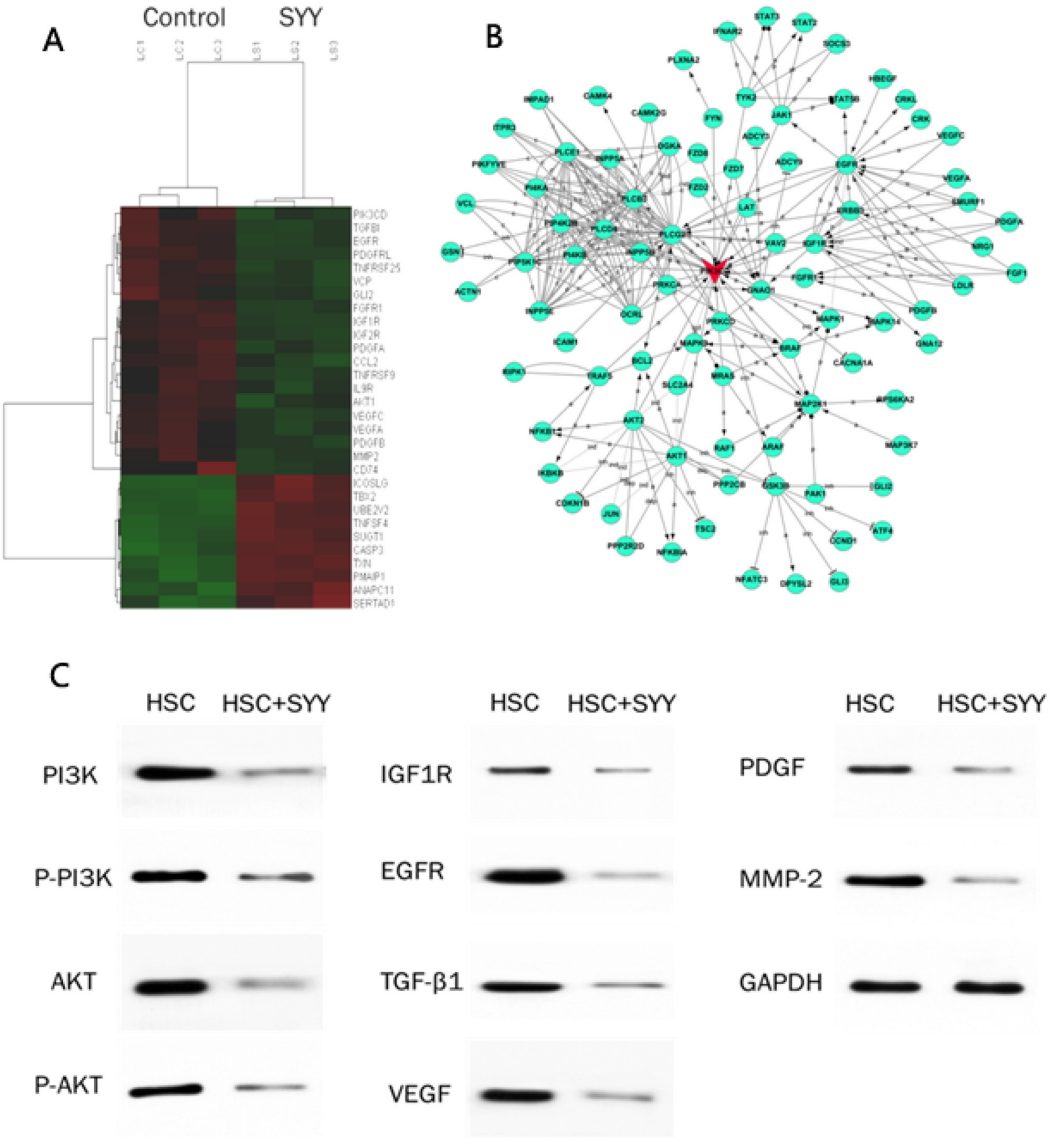
1205 up-regulated and 1323 down-regulated gene were detected by Gene Microarray analysis, and the most relevant data are shown **A.** Analysis of differential gene expression and signaling pathways indicated that PI3K was the key gene in the altered gene profiles **B.** Western blots analysis showed that PI3K, pPI3k, AKT, pAKT, IGF1R, EGFR, TGF-β, VEGF, PDGF and MMP-2 were all down-regulated following SYY treatment **C.**

There was difference between gene expression profiles of SYY treated LX2 cells and those of the treated controls. There were 1205 up-regulated genes and 1323 down-regulated genes following treatment of LX2 with SYY. Analysis of differential gene expression and signaling pathways indicated that PI3K was the key altered gene. The Western blot analysis of proteins in SYY treated LX2 cells revealed that PI3K related signaling factors were all significantly down-regulated, including PI3K, AKT, pAKT, IGF1R, EGFR, TGF-β1, VEGF, PDGF and MMP-2. (Figure 6A, 6B, 6C). Further, ELISA method showed that was significant decreased secretion of cytokines and other factors which were regulated by PI3K/AKT signaling after SYY treatment in LX2, such as TGFβ1 (CM-nSYY 32500 ± 143.5 *vs*. CM-SYY 26350 ± 127.3, *P* < 0.0001), MMP-2 (CM-nSYY 141500 ± 15710 *vs*. CM-SYY 92100 ± 951.2, *P* = 0.0350), HGF (CM-nSYY 9382 ± 314.5 *vs*. CM-SYY 3511 ± 349.2, *P* = 0.0003) and VEGF (CM-nSYY 1046 ± 89.40 *vs*. CM-SYY 664.6 ± 29.14, *P* = 0.0154). The additional cytokines altered significantly after SYY treatment of LX2 cells, were IL-10 (CM-nSYY 95.42 ± 1.379 *vs*. CM-SYY 134.7 ± 1.42, *P* < 0.0001), IL-12 (CM-nSYY 40.81 ± 2.52 *vs*. CM-SYY 134.7 ± 1.42, *P* < 0.0001), IL-2 (CM-nSYY 28.48 ± 1.04 *vs*. CM-SYY 81.97 ± 0.66, *P* < 0.0001), PDGF (CM-nSYY 6.02 ± 0.43 *vs*. CM-SYY 2.14 ± 0.88, *P* = 0.0161), EGF (CM-nSYY 2.97 ± 0.15 *vs*. CM-SYY 2.52 ± 0.16, *P* < 0.0001) and TNF-α (CM-nSYY2.82 ± 0.44 *vs*. CM-SYY 4.40 ± 0.56, *P* = 0.0897) (Table 2).

**Table 2:** Cytokine secretion of aHSCs after treatment with SYY

Cytokine	CM-nSYY (pg/ml)	CM-SYY (pg/ml)	*P* value
TGF-β1	32500 ± 143.5	26350 ± 127.3	< 0.0001
MMP2	141500 ± 15710	92100 ± 951.2	= 0.0350
IL-10	95.42 ± 1.379	134.7 ± 1.427	< 0.0001
IL-12	40.81 ± 2.524	89.99 ± 1.022	< 0.0001
IL-2	28.48 ± 1.0448	81.97 ± 0.6629	< 0.0001
PDGF	6.020 ± 0.4255	2.137 ± 0.8838	= 0.0161
EGF	2.975 ± 0.1520	2.552 ± 0.1587	< 0.0001
VEGF	1046 ± 89.4	664.6 ± 29.14	= 0.0154
HGF	9382 ± 314.5	3511 ± 349.2	= 0.0003
TNF-α	2.815 ± 0.4392	4.398 ± 0.5574	=0.0897

## DISCUSSION

Approximately 90% of HCC cases arise in patients with cirrhosis, which clearly indicates that liver fibrosis is strongly associated with the development of HCC [33]. HSCs are known to be one of the key cell types that contribute to the pathogenesis of liver fibrosis. It is well known that HSCs are activated in response to liver damage and trans-differentiate into mesenchymal fibroblasts (MFs), which play a key role in causing hepatic fibrosis. HSCs can also infiltrate the stroma of liver tumors as CAFs and further promote HCC cell proliferation and metastasis. It has been postulated that multiple signaling mechanisms may regulate aHSCs behavior which may induce changes in the hepatic tumor phenotype [34, 35]. A recent study has reported that aHSCs interact with hepatoma cells *via* secretion of cytokines, extracellular matrix (ECM)-mediated interactions and direct cell-to-cell contacts [36]. We previously reported that SYY indirectly attenuated hepatoma cell invasiveness and pulmonary metastasis through down-regulation of cytokines secreted by aHSCs [11]. However, the ability of SYY to reverse liver fibrosis and mechanisms regulating HCC inhibition remains largely unknown. Therefore, we chose a mouse model with a cirrhosis background to determine if SYY could attenuate hepatic fibrosis, block the cross-talk between aHSCs and HCC and indirectly influence the malignant potential of hepatoma cells.

Accumulating evidence supports the concept that aHSCs are the main matrix-producing cells in the process of liver fibrosis in response to liver damage [4, 5]. Numerous studies have shown that aHSCs up-regulate the gene expression of ECM components, matrix-degrading enzymes and respective inhibitors, resulting in ECM remodeling and accumulation [37]. In the present study, we established a stable mouse model with a fibrosis background using chronic subcutaneous injection of CCL_4_. We found that the severity of hepatic fibrosis was increased by enhancing particles on the liver surface, and there was liver volume with the prolonged treatment of CCL_4_. As expected, H&E and Sirius staining showed continuous collagen accumulation following CCL_4_ treatment. We also observed up-regulated α-SMA expression following CCL_4_ treatment. An increasingly complex interplay between various cell types of the liver, stellate cells with tumor cells had now become apparent. [38, 39]. Therefore, based on the previous reports regarding nude mouse model with fibrosis as mentioned above, we further established a similar model bearing orthotopic xenograft in mice with fibrosis background.

The development of distant metastasis requires the invasion of cancer cells from the primary tumor into the surrounding tissue. To acquire such invasive abilities, cancer cells must undergo several phenotypic changes. aHSCs, is one of the most important members in the tumor microenvironment, which plays a critical role in modulating such phenotypic changes. Amann *et al*., [12] demonstrated that conditioned medium (CM) collected from aHSCs could promote proliferation and migration of HCC cells. There are many cytokines secreted by aHSCs that are associated with tumor invasion and metastasis, including HGF, EGF, IL-1, IL-2, IL-6, MMP-2, MMP-9, TGF-β, TNF-α, VEGF and members of the Wnt signaling family [9, 39, 40]. Studebaker *et al*., [10] found soluble IL-6 produced by tissue-specific fibroblasts could promote growth and invasion of breast cancer cells, which could be inhibited by the removal or inhibition of IL-6. Li *et al*., [41] found that Wnt2, secreted by tumor fibroblasts, promoted tumor progression in oesophageal cancer by activation of the Wnt/β-catenin signaling pathway. Recently, Jia *et al*., [11], found that SYY could attenuate hepatic cancer cell's invasive and metastatic capabilities by down-regulating cytokines secreted by aHSCs such as HGF, IL-6, TGF-β and VEGF *in vitro*. In the present study, we found that SYY could have an effect on fibrosis and also could block the cross-talk between aHSCs and HCC.

SYY is a Chinese herbal medicine consisting of five herbs. Some components of SYY, such as Tanshinone and *Astragalus*saponins, have demonstrated efficacy in treatment of malignancies [42, 43]. We have previously shown that SYY could effectively inhibit tumor growth and metastasis, reverse the molecular changes consistent with endothelial to mesenchymal transition and increase survival in a HCC nude mouse model [30, 31]. In the present study, SYY inhibited liver cancer, reduced associated fibrosis and prolonged survival in the subcutaneous tumor model with fibrosis background. In addition, using an *in vitro* model, we also demonstrated that hepatoma cells treated by CM from SYY pretreated aHSCs had reduced malignant potential, with altered gene expression profile consisting of 1205 up-regulated and 1323 down-regulated genes. We found that PI3K was the key gene present in this altered gene profile. Further, protein analysis showed that the PI3K related markers such as PI3K AKT, pAKT, IGF1R, EGFR, TGF-β1, VEGF, PDGF and MMP-2 were all down-regulated significantly after SYY treatment in LX2.

In the present study, we demonstrated that SYY could reduce liver cirrhosis and attenuate hepatic cancer cell invasiveness and metastasis through the down-regulation of cytokines secreted by activated hepatic stellate cells. Our findings suggest that in future traditional Chinese medicines, such as SYY, may provide novel therapeutic approaches targeted to reduce liver cirrhosis and prevent liver cancers.

## MATERIALS AND METHODS

### Cell lines and animals

A highly metastatic human hepatocellular carcinoma cell line (HCCLM3), which originated from MHCC97H, was established as previously described [26, 27]. Hep3B from American Type Culture Collection was purchased from the Chinese Academy of Science. Male BALB/c nu/nu mice (aged 4–6 weeks and weighing approximately 20 g) were obtained from the Chinese Academy of Science and maintained under standard pathogen-free conditions. The animal use protocol was approved by the Shanghai Medical Experimental Animal Care Commission.

### Regents and antibodies

Antibodies used for immunofluorescence, immunoblotting and/or immunohistochemistry were as follows: rabbit antihuman monoclonal α-smooth muscle actin (α-SMA) (Abcam, Cambridge, MA, USA), rabbit anti-human monoclonal PI3K (Epitomics, Burlingame, CA, USA), rabbit antihuman monoclonal AKT (CST), rabbit antihuman monoclonal phosphorylated (p)-AKT (CST, Danvers, MA, USA), rabbit antihuman monoclonal insulin like growth factor-1 receptor (IGF1R) (Abcam), rabbit antihuman monoclonal epidermal growth factor receptor (EGFR) (Abcam), rabbit antihuman monoclonal transforming growth factor-β1 (TGF-β1) (Abcam), rabbit antihuman monoclonal vascular endothelial growth factor (VEGF) (Epitomics), rabbit antihuman monoclonal platelet derived growth factor (PDGF) (Epitomics), rabbit antihuman monoclonal MMP-2 (Epitomics) and rabbit antihuman monoclonal proliferating cell nuclear antigen (PCNA) (Epitomics).

### Characterization and preparation of herbal extracts

The Chinese herbal medicine formula for SYY is a dietary compound approved by the Chinese State Food and Drug Administration (Grant No. G20070160) that includes five Chinese medicinal herbal extracts with a finger print of the following proportions (w/w): Salviamiltiorrhiza Bge, 14.3%; Astragalus membranaceus Bge, 14.3%; Lycium barbarum L, 23.8%; Crataegus pinnatifida Bge, 23.8% and Trionyx sinensis Wiegmann, 23.8% (all from China) [11]. SYY, with the same batch number (#20110401), used *in vitro* was produced by Shanghai Fang Xin Pharmaceutical Technology Co., Ltd. (Shanghai, China). The 800 mg/ml SYY preparation was sterilized twice by filtration with 0.22 μm filter. (Millipore, Billerica, MA, USA). It was further prepared for *in vitro* use as described previously [32].

### Preparation of conditioned media (CM)

The aHSCs were cultured in high glucose Dulbecco's modified eagle medium (DMEM) containing 10% (v/v) FBS in T_25_ flasks (1 × 10^5^ cells) as previously described [11]. The next day, cultures were removed and 12 ml fresh serum-free DMEM medium with SYY sample (2 mg/ml) was added to SYY group, and fresh DMEM medium was added to control sample (no SYY). 24 h later, the cultures were centrifuged at 800 rpm and the supernatants were collected. All cell culture reagents were purchased from Invitrogen.

### Lactate dehydrogenase (LDH) cytotoxicity assay

A cytotoxicity detection kit PLUS (Roche) that quantitates cytotoxicity by measuring lactic dehydrogenase (LDH) activity released from damaged cells. The aHSCs were cultured and removed from the plates with trypsin digestion when the cell density reached 80% confluence. The cells were then counted and pipetted into 96-well plates at 1000 cells/well. The same plate contained background controls (medium only), low controls (spontaneous LDH release), high controls (maximum LDH release) and experimental samples (2 mg/ml SYY) which were prepared according to the manufacturer's instructions. These plates were incubated in an incubator at 37°C in 5% CO2 for 4, 8, 12, 24, 48 and 72 h. Results were expressed as the mean absorbance of wells in groups measured at 492 nm. Cytotoxicity (%) was calculated using the equation: (experimental value − low control) / (high control − low control) × 100%.

### Cell proliferation and colony formation assay

aHSCs were cultured in 96-well plates (3 × 10^3^ cells/well) and exposed to SYY at increasing concentrations for 24, 48, 72 and 96 h. Cell proliferation assays were carried out with the Cell Counting Kit 8 (CCK8; Dojindo) and absorbance (OD) of each well noted at 450 nm. For colony formation assays, LM3 and Hep3B cells were cultured in 6-well plates (3 × 10^3^ cells/well) and cultured with DMEM and CM from aHSCs with or without SYY (2 mg/mL). Culture medium was replaced every 3 days, and the colonies were fixed with ice-cold 4% paraformaldehyde 14 days after the initiation of treatment. Cells were stained with Giemsa (Sigma) and photographed.

### Cell invasion assays

Cell invasion of HCCLM3 and Hep3B cell lines were assessed by transwell assays (Boyden chambers; Corning). Briefly, 80 μl matrigel (BD Biosciences) was added to the upper chamber 6 h prior to seeding the cells on the membrane. Subsequently, 6 × 10^4^ cells were seeded into the upper chamber of each well of 24-well plates containing 8.0-μm pore size membranes. Serum-free DMEM, with or without SYY and aHSCs CM with or without SYY were added to the lower chamber of each well. After 48 h, cells that had crossed over to the underside of the membrane were visualized by staining with Giemsa (Sigma), and photographed at a magnification of 200 x.

### Immunofluorescence and western blot analysis

The expression of markers PCNA and α-SMA were determined by immunofluorescence. Fibrotic hepatic tissues were sliced, fixed, permeabilized, blocked and incubated with primary monoclonal antibodies overnight at 4°C. Slides were washed and incubated with anti-rabbit Cy3-conjugated secondary antibody (Jackson). Cells were counterstained with 4–6-diamidino-2-phenylindole (DAPI) to visualize cell nuclei and detected by fluorescence microscopy (Olympus). The concentration of protein extracted from aHSCs and aHSCs–SYY was determined using BCA Protein Assay Kit (Beyotime). Western blot analysis of PI3K, AKT, pAKT, IGF1R, EGFR, TGF-β1, VEGF, PDGF and MMP-2 protein expression was performed according to the manufacturer's instructions.

### Expression of DNA microarray and analysis

DNA microarrays were used to evaluate changes in gene expression profiles of aHSCs after SYY treatment. Total RNA extracted from SYY treated aHSCs and control cells were used for DNA microarray analysis. Microarray analysis of three independent samples was performed using 4 × 44K human Genome Array chips (Taiwan Phalanxbiotech Gene Expression Profiling Microarray), according to the manufacturer's instructions. Data analysis was performed using Feature Extraction and bioinformatics software GENESPRING ver12.0

### Establishment of animal model and treatment procedure

#### Establishment of the nude mouse with liver fibrosis

Male BALB/c nu/nu mice (4–6 weeks old; *n* = 30) received subcutaneous injection of 5 μl/g carbon tetrachloride (CCl_4_/olive oil: 50:50) weekly. From the fourth week of starting injection, 6 nude mice were sacrificed every two weeks by spine dislocation to identify the degree of fibrosis by hematoxylin and eosin (H&E) and Sirius staining. Further, α-SMA was detected using immunohistochemistry till the tenth week.

#### Establishment of the nude mouse with liver fibrosis bearing orthotopic xenograft

Male BALB/c nu/nu mice (4–6 weeks old; *n* = 20) bearing orthotopic xenografts from HCCLM3 were randomly divided into four groups. Three days later, two groups were subcutaneously injected with CCl_4_ (5 μl/g/week), the other two were treated with equal dose of olive oil. At the same time, one group chosen from the CCl_4_ treated group and the other group from olive oil groups were administered SYY through the drinking medium (2 g/kg/day). After 6 weeks, all nude mice were sacrificed and tumor volume and the degree of fibrosis were evaluated.

#### Establishment of the nude mouse bearing subcutaneous tumor with fibrosis background

Male BALB/c nu/nu mice (4–6 weeks old; *n* = 20) were divided into four groups. Each group of nude mice were subcutaneously injected with different number (1 × 10^6^, 5 × 10^5^, 1 × 10^5^, 5 × 10^4^) of highly metastatic HCCLM3 cells (0.1 ml). After 12 weeks, following the mice sacrifice the relationship between tumor growth and the number of tumor cells was evaluated.

Male BALB/c nu/nu mice (4–6 weeks old; *n* = 25) were divided into five groups. Four groups of nude mice were subcutaneously injected with mixed cells (different number of HCCLM3 cells (0.1ml, 1 × 10^6^, 5 × 10^5^, 1 × 10^5^, 5 × 10^4^,) and human hepatic stellate cells (0.1 ml LX2, 1 × 10^5^). The other group was just injected with LX2 cells (2 × 10^5^, 0.2 ml). After 12 weeks, all the mice were sacrificed by cervical dislocation and the subcutaneous tumor formation was evaluated.

Male BALB/c nu/nu mice (4–6 weeks old; *n* = 6) were divided into two groups. These mice were subcutaneously injected with mixed cells (HCCLM3-GFP cells (5 × 10^5^) and LX2 cells (5 × 10^5^). One group was treated with SYY for 6 weeks, the other group without intervention. After 6 weeks, the tumor volume was evaluated. Lung metastases were also evaluated by fluorescence microscopy. Then taking two groups of mice, treated in the same way, survival status was determined.

### Statistical analysis

Statistical analysis was carried out using SPSS software version 10.0 for Windows software (SPSS, Chicago, IL, USA). As all groups showed normal distribution, group differences were analyzed using parametric statistical methods, paired independent sample *t*-tests following one-way ANOVA. Data were presented as mean ± standard deviation, *P* < 0.05 was considered statistically significant.

## SUPPLEMENTARY FIGURE


