# Supplementary material for: The herbal compound Songyou Yin (SYY) inhibits hepatocellular carcinoma growth and improves survival in models of chronic fibrosis via paracrine inhibition of activated hepatic stellate cells
Source: Oncotarget. 2015 Oct 22;6(37):40068–80. doi: 10.18632/oncotarget.5313 (PMC4741880; doi:10.18632/oncotarget.5313)
Supplement: Supplementary file 1 [file oncotarget-06-40068-s001.pdf]

SUPPLEMENTARY FIGURE

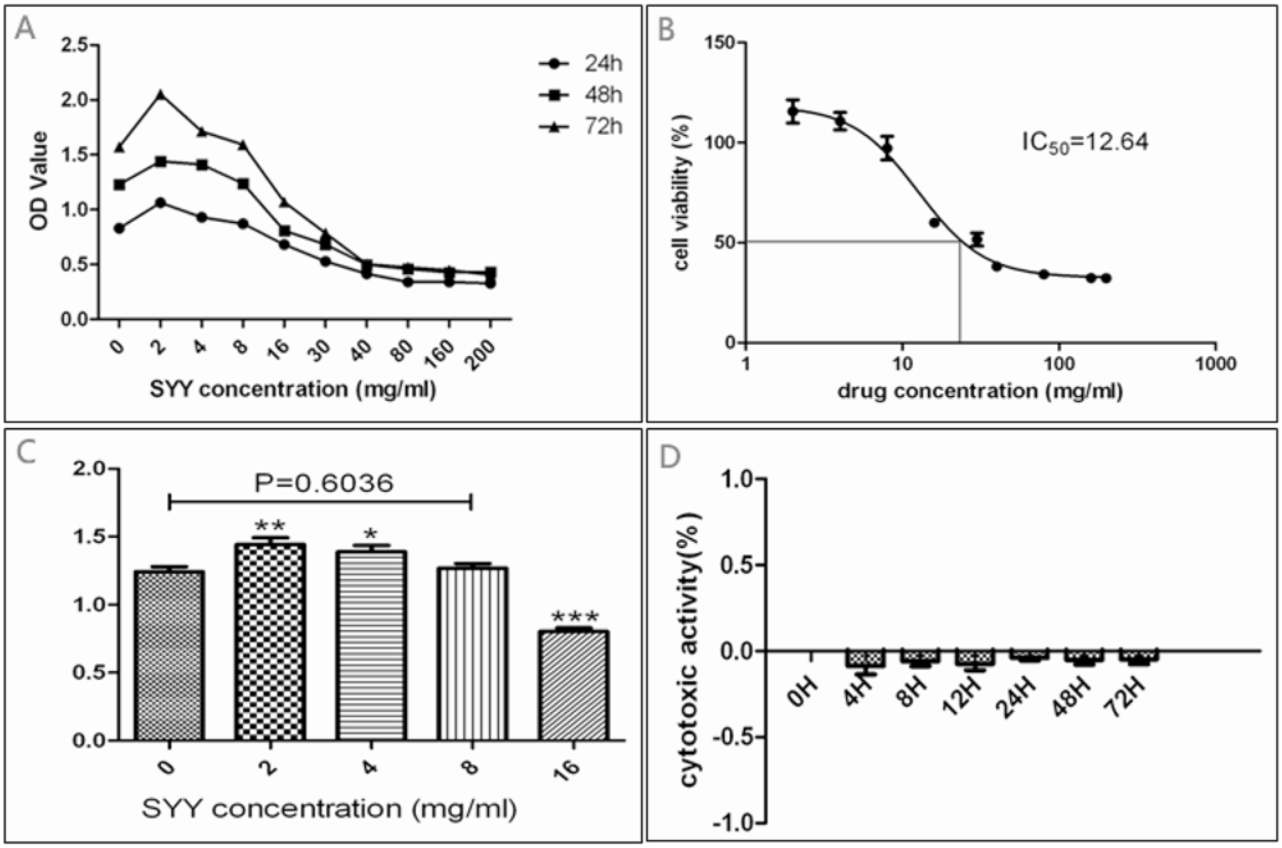

Supplementary Figure S1: SYY inhibited proliferation in LX2 cells with increasing drug concentration, especially when the concentration reached 16 mg/ml A. The IC50 was 12.64 mg/ml which was examined by CCK8 assay **B.** SYY (8 mg/ml) exhibited no significant growth inhibitory effect on LX2 cells **C.** SYY (8 mg/ml) exhibited no significant cytotoxicity in LX2, as there was no statistically difference in the relative emission of LDH **D.**
